# INVOLVING COMMUNITY GOVERNMENTAL ORGANIZATIONS IN THE ESTABLISHMENT OF DEMENTIA-FRIENDLY COMMUNITIES IN BEIJING

**DOI:** 10.1093/geroni/igz038.1666

**Published:** 2019-11-08

**Authors:** Yu Liu, Hexin Zhao, Hong Guo

**Affiliations:** 1 China Medical University, Shenyang, Liaoning, China; 2 Beijing Hospital, beijing, Beijing, China (People’s Republic); 3 Beijing Traditional Chinese Medicine University School of Nursing, Beijing, Beijing, China (People’s Republic)

## Abstract

China has about 20% of the world’s total dementia population. Since most elders with dementia are living at home with care by family members, communities are fundamental support resources for families as well as patients. Dementia-friendly community initiatives aim to empower families with dementia and increase their social inclusion. Within the Chinese political context, the community level governmental organization called Ju-wei-hui has played a key role in community engagement. Within this context, a Community Based Participatory Action Research (CBPAR) process is utilized to increase public awareness on dementia and caring strategies. Our team collaborated with 15 Ju-wei-hui offices across Beijing to design a series of courses and teaching modules together. Five hundred community residents participated and positively evaluated the project. A major finding is that CBPAR could be an effective strategy to develop dementia-friendly communities across China.

